# Conservation and Expression Patterns Divergence of Ascorbic Acid d-mannose/l-galactose Pathway Genes in *Brassica rapa*

**DOI:** 10.3389/fpls.2016.00778

**Published:** 2016-06-02

**Authors:** Weike Duan, Jun Ren, Yan Li, Tongkun Liu, Xiaoming Song, Zhongwen Chen, Zhinan Huang, Xilin Hou, Ying Li

**Affiliations:** ^1^State Key Laboratory of Crop Genetics and Germplasm Enhancement, Key Laboratory of Biology and Germplasm Enhancement of Horticultural Crops in East China, College of Horticulture of Nanjing Agricultural UniversityNanjing, China; ^2^Center of Genomics and Computational Biology, College of Life Sciences, North China University of Science and TechnologyTangshan, China

**Keywords:** *Brassica rapa*, conservative, D-mannose/L-galactose pathway, expression divergence, evolutionary pattern

## Abstract

Ascorbic acid (AsA) participates in diverse biological processes, is regulated by multiple factors and is a potent antioxidant and cellular reductant. The D-Mannose/L-Galactose pathway is a major plant AsA biosynthetic pathway that is highly connected within biosynthetic networks, and generally conserved across plants. Previous work has shown that, although most genes of this pathway are expressed under standard growth conditions in *Brassica rapa*, some paralogs of these genes are not. We hypothesize that regulatory evolution in duplicate AsA pathway genes has occurred as an adaptation to environmental stressors, and that gene retention has been influenced by polyploidation events in Brassicas. To test these hypotheses, we explored the conservation of these genes in Brassicas and their expression patterns divergence in *B. rapa*. Similar retention and a high degree of gene sequence similarity were identified in *B. rapa* (A genome), *B. oleracea* (C genome) and *B. napus* (AC genome). However, the number of genes that encode the same type of enzymes varied among the three plant species. With the exception of GMP, which has nine genes, there were one to four genes that encoded the other enzymes. Moreover, we found that expression patterns divergence widely exists among these genes. (i) *VTC2* and *VTC5* are paralogous genes, but only *VTC5* is influenced by *FLC*. (ii) Under light treatment, *PMI1* co-regulates the AsA pool size with other D-Man/L-Gal pathway genes, whereas *PMI2* is regulated only by darkness. (iii) Under NaCl, Cu^2+^, MeJA and wounding stresses, most of the paralogs exhibit different expression patterns. Additionally, GME and GPP are the key regulatory enzymes that limit AsA biosynthesis in response to these treatments. In conclusion, our data support that the conservative and divergent expression patterns of D-Man/L-Gal pathway genes not only avoid AsA biosynthesis network instability but also allow *B. rapa* to better adapt to complex environments.

## Introduction

l-Ascorbic acid (AsA, the reduced form of VTC) is an essential metabolite in plants and animals. Plants can synthesize this indispensable vitamin for themselves, but humans and some other animals must obtain AsA from fruits and vegetables (Nishikimi et al., 1994). In plants, AsA is not only an important nutrient source for humans but also an important antioxidant and redox buffer that has critical roles in metabolism by responding to abiotic stresses and pathogens (Pavet et al., 2005; Zhang, 2012). In addition, plants require AsA as a cofactor for growth regulation, cell wall synthesis (de Pinto and De Gara, 2004), stomatal closure and flowering time (Chen and Gallie, 2004; Kotchoni et al., 2009).

Significant progress has been made in understanding the AsA biosynthesis pathways and their components in plants. AsA was proposed to be synthesized via D-mannose and L-galactose in a pathway called the D-Mannose/L-Galactose (D-Man/L-Gal; Smirnoff–Wheeler) pathway (Wheeler et al., 1998). In addition, several other pathways of AsA biosynthesis have been identified in plants, such as the L-gulose, D-galacturonate and *myo*-inositol pathways (Valpuesta and Botella, 2004). However, additional biochemical and molecular genetic evidence exists suggesting that the D-Man/L-Gal pathway is the main route of AsA biosynthesis in plants. Conklin et al. (2000) screened *Arabidopsis* mutants with defective AsA synthesis and identified 5 loci (*VTC1, VTC2, VTC3, VTC4* and *VTC5*) related to the AsA D-Man/L-Gal pathway (Conklin et al., 2000). *VTC1* encodes the AsA biosynthetic enzyme GMP (Conklin et al., 1999), *VTC2* and *VTC5* encode GGP (Dowdle et al., 2007; Linster et al., 2007), and *VTC4* encodes GPP (Conklin et al., 2006; Laing et al., 2007). All of these genes are essential for AsA biosynthesis, as evidenced by experiments performed in *vtc* mutants. It is well documented that light regulates AsA biosynthesis; *Arabidopsis* photomorphogenic factor COP9 signalosome subunit 5B interacts with *VTC1* and modulates AsA synthesis and the response to salt stress (Wang et al., 2013). A light-responsive *cis*-element has recently been identified in the promoter of the *VTC2* gene (Gao et al., 2011). Furthermore, *VTC5* is one of putative targets of *FLOWERING LOCUS C* (*FLC*), which plays a key role in the timing of the initiation of flowering and potentially regulates genes that function in many developmental pathways (Deng et al., 2011). In addition, AsA-related genes play important roles in the responses to multiple stresses. Zhang et al. (2012) reported that Ethylene responsive factor 98 (ERF98) in *Arabidopsis* transcriptionally activates *VTC1* gene expression in AsA biosynthesis and contributes to enhanced salt tolerance (Zhang et al., 2012). In summary, stress can lead to ROS production, whereas higher AsA levels benefit plants by helping to eliminate the accumulated ROS. Thus, the AsA D-Man/L-Gal pathway is essential for plant growth, participates in stress resistance, and appears to be involved in flowering time control.

AsA D-Man/L-Gal genes are present in both land plants and green algae, suggesting that this pathway evolved in the last common ancestor of green plants (Duan et al., 2015). The higher plant lineages have undergone polyploidization during their long evolutionary history. Based on the gene balance hypothesis, several genes with products that participate in metabolic networks or in transcriptional or signaling networks have been preferentially retained to avoid network instability caused by the loss of one member (Lynch and Conery, 2000; Birchler and Veitia, 2007). Compared with the neighboring genes, the core eukaryotic genes and randomly selected genes from the microsyntenic regions corresponding to the AsA-related genes, all of the AsA-related genes have been preferentially retained since the divergence of *Brassica rapa* from the last common ancestor with *Arabidopsis thaliana*. Additionally, the D-Man/L-Gal pathway genes are highly conserved and there are no significant differences in the gene numbers among selected representative plant species (Duan et al., 2015).

During evolution, gene duplication has provided plant genes an opportunity to transform their form and functions to adapt to environmental changes (Rensing, 2014). After duplication, some of the gene pairs have a brief life, or only one copy is maintained. In contrast, some gene pairs survive after the duplication require for adaptive evolution, and their subsequent divergence provide raw genetic resources (subfunctionalization) or play a central role in the evolution of novel gene functions (neofunctionalization) (Flagel and Wendel, 2009). Brassicaceae is a large eudicot family including the model plant *A. thaliana* and the ‘U’s triangle Brassicas crops (Nagaharu, 1935), which are major contributors to the human diet. The triangle consists of the three diploid species, *B. rapa* (A genome, *n* = 10), *B. nigra* (B genome, *n* = 8), and *B. oleracea* (C genome, *n* = 9). Furthermore, they have formed three amphidiploid species, including *B. juncea* (AB, *n* = 18), *B. napus* (AC, *n* = 19), and *B. carinata* (BC, *n* = 17) by hybridization. These species share a complex history with *A. thaliana* and then experienced an additional whole-genome triplication (WGT) event 13–17 million years ago (Wang et al., 2011; Cheng et al., 2013). *B. rapa* includes several subspecies, such as Chinese cabbage (*B. rapa* ssp. *pekinensis*) and NHCC (*B. rapa* ssp. *chinensis*; Pak-choi), which are economically significant vegetable crops in Asia. Moreover, NHCC is one of the most important vegetables in south China. The AsA content not only is essential for plant growth but also is an important index to evaluate the quality of the vegetables. However, in *B. rapa*, increased AsA content does not correlate with the number of AsA paralogs that are expressed after the WGT event (Duan et al., 2015). What happened to the duplicated genes in the AsA D-Man/L-Gal pathway after polyploidization remains an open question. *B. rapa, B. oleracea*, and *B. napus* have recently been sequenced, and their genomes have been assembled (Wang et al., 2011; Chalhoub et al., 2014; Liu S. et al., 2014), thus providing good raw data to study the evolutionary patterns of duplicated genes.

This work explored the conservation and expression patterns divergence of AsA D-Man/L-Gal pathway genes and their regulatory mechanism under multiple treatments. Specifically, our study (i) compared the retention and conservation of AsA D-Man/L-Gal pathway genes in the Brassica A and C genomes; (ii) identified the number of genes that encode the same type of enzymes and determined the structural divergence in the subclades of GMPs; (iii) identified the evolutionary history of *VTC2* and *VTC5* through 5 eudicots and 1 basal angiosperm; (iv) showed that AsA D-Man/L-Gal pathway genes are regulated by light in *B. rapa*; (v) analyzed the co-expression and differential expression patterns of AsA D-Man/L-Gal pathway genes involved in response to multiple abiotic stresses in *B. rapa*; and (vi) explored the different expression patterns of homologous genes.

## Materials and Methods

### Sequence Retrieval

The sequences of the *B. rapa* AsA D-Man/L-Gal pathway genes were retrieved from the *Brassica* database (BRAD^1^), according to previous reports (Duan et al., 2015). The genome data set of *B. oleracea* was downloaded from Bolbase^2^ (Liu S. et al., 2014), and that of *B. napus* was downloaded from the *Brassica napus* Genome Browser^3^ (Chalhoub et al., 2014). The gene sequences from *Vitis vinifera, Carica papaya, Populus trichocarpa*, and *Amborella trichopoda* were downloaded from Phytozome v9.1^4^ (Goodstein et al., 2012). The sequences of the *B. oleracea* and *B. napus* homologs to these AsA-related genes in *B. rapa* were identified through a BLASTp search (E-value 1e ≤ 20, identity ≥ 40%) (Duan et al., 2015). The GGP homologs in *V. vinifera, C. papaya, P. trichocarpa*, and *A. trichopoda* genomes were identified in the same way. Then, we verified these sequences in the NCBI database^5^.

### Synteny Analysis of AsA D-Man/L-Gal Pathway Genes

The position of each AsA D-Man/L-Gal pathway gene on the syntenic blocks was verified by searching for homologs among *A. thaliana* and the LF, MF1 and MF2 subgenomes of *B. rapa, B. oleracea*, and *B. napus* using BRAD^6^ (last accessed January 8, 2015) (Cheng et al., 2012). Conservation of chromosomal synteny around the GGPs in *A. thaliana, A. trichopoda, C. papaya, P. trichocarpa, V. vinifera*, and *B. rapa* was evaluated with CoGe^7^. An in-house Perl program was used to draw the syntenic diagram.

The potential duplicated genes in the *B. rapa, B. oleracea* and *B. napus* genomes were identified using MCScanX^8^ (Wang et al., 2012). The resulting blast hits were incorporated, along with the chromosome coordinates of all protein-coding genes, as an input for MCScanX and classified into segmental, tandem, proximal and dispersed duplications under the default criteria.

The set of core eukaryotic genes and a set of randomly selected genes from the microsyntenic regions corresponding to the AsA D-Man/L-Gal pathway genes and a set of genes flanking the *A. thaliana* AsA D-Man/L-Gal pathway genes (10 on either side) were established according our previous study (Duan et al., 2015).

### Multiple Sequence Alignments, Phylogenetic Relationship and Conserved Motif Analyses

The amino acid sequence alignments of the full-length protein sequences of the AsA-related genes were aligned with the MUSCLE program using the default parameters (Nei, 1996; Edgar, 2004). The phylogenetic trees were then constructed with the ML method in each analysis by using MEGA 5.2 (Tamura et al., 2011). The confidence level of the monophyletic clade was estimated using a bootstrap analysis of 1,000 replicates.

The putative AsA-related protein sequences used for the phylogenetic analysis were detected by MEME program version 4.9.0 to analyze the possible conserved motifs by using default parameters (Bailey et al., 2009), except for the following parameters: the maximum number of motifs was set to 10 and the optimum motif width was set to ≥10 and ≤100.

### Plant Material, Growth Conditions and Stress Treatments

One of the NHCC cultivars (*B. rapa* ssp. *chinensis* cv. *suzhouqing*) was used for the experiments. The seeds were washed with distilled water and germinated on moist filter paper in a 25°C incubator in the dark for 2 days. The germinated seeds were grown in pots containing soil consisting of a vermiculite mixture (3:1) in the greenhouse of Nanjing Agricultural University. The controlled-environment growth chamber was programmed for 75% humidity, light 16 h/25°C and dark 8 h/20°C. Light was set as 100 μmol m^-2^ s^-1^. Seedlings at the five-leaf stage were used for the subsequent experiments: (i) light/darkness treatment: the analysis started after 8 h of darkness (corresponding to the time of starting the light period), and the plants were then exposed to continuous dark or were moved into the light at 100 μmol m^-2^ s^-1^ for 24 h. Under the light/darkness treatment, the temperature was set to 25°C. Samples were collected every 3 h. (ii) Simulated vernalization treatment: the plants were placed at 4°C for 50 days. The controlled-environment growth chamber was programmed for 75% humidity, light 16 h and dark 8 h. Samples were collected at each time point (0, 1, 3, and 5 and then every 5 days). (iii) Multiple-stress treatments: for acclimation, some plants were cultured in 1/2 Hoagland’s solution, pH 6.5, in plastic containers (Jensen and Bassham, 1966). The controlled-environment growth chamber was set as described above (light 16 h/25°C and dark 8 h/20°C). After 1 week of acclimatization, plants were cultured in the following five treatments: (1) Control, (2) 100 μM CuSO_4_, (3) 1 mM MeJA, (4) 100 μM NaCl and (5) wounding simulation (the leaf was wounded via two 1 cm diameter holes on each side of the main vein with a sterile hole punch). For these treatments, samples were collected at each time point (0, 6, 12, 24, and 48 h). For all treatments, there were three biological replicates. In addition, the T_2_ *BracFLC1* transgenic *A. thaliana* plants from the study by Liu et al. (2012) were also used in this study (Liu et al., 2012). All samples were frozen in liquid nitrogen and stored at -70°C until further analysis.

### Analyses of AsA and Total-AsA

The AsA and T-AsA levels were analyzed according to the procedure described by Melino et al. (2009), with the slight modifications described by Ren et al. (2013). The samples (0.2 g of leaf tissue) were homogenized in 1.6 mL of 0.1% (w/v) pre-cooled oxalic acid and then centrifuged at 12,000 *g* for 20 min at 4°C. The supernatants were filtered through a 0.45-mm membrane syringe filter. Finally, the filtrate was collected for HPLC assays of AsA at a wavelength of 245 nm. T-AsA was analyzed by adding 20 mL of dithiothreitol (20 mg/L) to 20 mL of the filtrate and incubating the mixtures for 15 min in the dark. The reaction solution was also used for HPLC assays of T-AsA at a wavelength of 245 nm.

### RNA Isolation and qRT- PCR

Leaf samples (0.1 g) were obtained from control and from treated plants and were subjected to total RNA extraction with an RNA kit (RNAsimply total RNA Kit, Tiangen, Beijing, China) according to the manufacturer’s instructions. Five micrograms of each sample was reverse transcribed into cDNAs using a PrimeScript RT reagent Kit (TaKaRa). The gene-specific primers for the D-Man/L-Gal pathway genes that were used to detect transcripts were designed according to a previous study by Ren et al. (2013) (Supplementary Table S1). Each qRT-PCR reaction (20 μL) contained a diluted cDNA sample as the template, 2× Power SYBR Green PCR Master Mix (Applied Biosystems) and 400 nM each of the forward and reverse gene-specific primers. The reactions were performed using a 7500 Fast Real-Time PCR System (Applied Biosystems) with the following cycling profile: 94°C for 30 s, followed by 40 cycles at 94°C for 10 s and then 58°C for 30 s. After the PCR was run, a melting curve (61 cycles at 65°C for 10 s) was generated to confirm the specificity of the amplification. Actin (Accession number: AF111812) was used as an internal control to normalize the expression level of the target genes among the different samples. The relative expression ratio of each gene was calculated using the comparative C_t_ value method (Heid et al., 1996). Gene expression was measured from at least three biological replicates (with three technical replicates per biological replicate).

### Pearson Correlation Coefficients (PCCs) Analyses

The PCCs for the AsA content and the transcript levels of AsA-related gene pairs were calculated using an in-house Perl script based on the transformed qRT-PCR data from the different treatments (Tang et al., 2013). In detail, the PCCs were calculated according to the relative expression trends at different treatment times, as previously described (Liu J. et al., 2014; Xu et al., 2015). The correlation was analyzed by using the R package, on the basis of the PCCs. The entire gene pairs whose PCCs were significant at a 0.05 significance level (*p*-value) were collected for a gene co-regulatory network analysis and the co-expressed networks were graphically visualized using Cytoscape version 3.1 based on the PCCs of these gene pairs (Shannon et al., 2003).

## Results

### Triplication and Fractionation of AsA D-Man/L-Gal Pathway Genes in Brassicas

To investigate the copy number variations of the AsA D-Man/L-Gal pathway genes in detail during the *Brassica*-specific WGT event in *B. rapa*, all 15 *A. thaliana* AsA D-Man/L-Gal pathway genes and three other sets were used. The sets included core eukaryotic genes and randomly selected genes from the microsyntenic regions corresponding to the *A. thaliana* AsA D-Man/L-Gal pathway genes and the neighboring genes flanking the AsA D-Man/L-Gal pathway genes. The number of AsA D-Man/L-Gal pathway genes that were completely lost in *B. rapa*, compared with the other three sets, was the lowest (6.7%), and the numbers (∼50%) of D-Man/L-Gal pathway and core eukaryotic genes that were retained as two or three copies were similar and were higher than those in the neighboring and randomly chosen gene sets (Supplementary Figure S1). Thus, the AsA D-Man/L-Gal pathway genes were preferentially retained in *B. rapa*.

**FIGURE 1:**
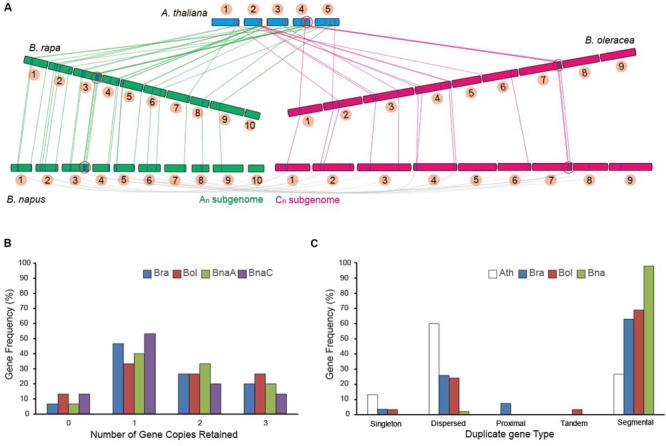
**Retention of homologous copies of the AsA D-Mannose/L-Galactose (D-Man/L-Gal) pathway genes in the syntenic regions in Brassicas and *Arabidopsis thaliana*.** The Brassicas in this study include *Brassica rapa, Brassica oleracea* and *Brassica napus*. **(A)** Syntenic alignments of AsA D-Man/L-Gal pathway genes between the model crucifer *A. thaliana, B. rapa, B. oleracea*, and *B. napus*. The link lines indicate the homologs. The red circles indicate the microsyntenic blocks. **(B)** Copy numbers of AsA D-Man/L-Gal pathway genes after genome triplication and fractionation in Brassicas. **(C)** Retention of homologous copies of AsA D-Man/L-Gal pathway genes in the three subgenomes (LF, MF1 and MF2) in Brassicas. LF: the least fractionated subgenome; MF1: moderate gene fractionation; MF2: the most fractionated gene.

To further investigate the retention of AsA D-Man/L-Gal pathway genes in other *Brassica* crops, the sequenced species *B. rapa* (AA), *B. oleracea* (CC) and *B. napus* (AACC; allopolyploidy) were used in this study. All of the 27 AsA D-Man/L-Gal pathway genes were identified in *B. rapa* (Duan et al., 2015). From the BLAST program and the syntenic analysis, 30 and 46 genes were identified in *B. oleracea* and *B. napus*, respectively (Supplementary Tables S2 and S3). Syntenic alignments of the AsA D-Man/L-Gal pathway genes between the model crucifers *A. thaliana, B. rapa, B. oleracea* and *B. napus* confirmed that triplication and fractionation had occurred in these three *Brassica* species (**Figure 1A**). With the exception of some *GMP* genes, the entire pathway genes were retained (Supplementary Table S3). The orthologous gene pairs of these genes in *B. rapa* and *B. oleracea* were also retained as homologous pairs in *B. napus*. The genes on the chromosomes of *B. rapa* and *B. oleracea* corresponded to the A_n_ and C_n_ subgenome chromosomes of *B. napus*, respectively (**Figure 1A**). Interestingly, the retained genes were in the same subgenomes (LF, MF1 and MF2) among the three *Brassica* species (Supplementary Table S3). Furthermore, certain genes (e.g., *PGI1, VTC2, GMP2* and *GDH*) in the microsyntenic blocks were retained together (**Figure 1A**).

We specifically compared the retention of AsA D-Man/L-Gal pathway genes in the AA genome (*B. rapa* and *B. napus* A_n_) and in the CC genome (*B. oleracea* and *B. napus* C_n_). The retention of AsA D-Man/L-Gal pathway genes after triplication and fractionation in Brassicas was not identical (**Figure 1B**). Similar copy numbers were found in the *B. rapa* and *B. napus* A_n_ genomes, but, owing to loss of the *GMP* genes on the syntenic blocks, there were two and three gene copies fewer in the *B. napus* C_n_ subgenome than in other *Brassica* genomes (Supplementary Table S3; **Figure 1B**). Overall, half (∼50%) of these genes were retained as two or three copies (**Figure 1B**) and more than 60% witnessed a segmental duplication event (**Figure 1C**), indicating that segmental duplication led to the expansion of AsA D-Man/L-Gal pathway genes after the WGT in Brassicas.

In this study, similar exon numbers were found in the orthologous gene pairs of *B. rapa, B. oleracea*, and *B. napus* (Supplementary Table S4). Furthermore, a pairwise protein sequence comparison among *A. thaliana, B. rapa, B. oleracea*, and *B. napus* showed high sequence identities >85% (between *A. thaliana* and *B. rapa*) and >95% (among the three *Brassica* species) via box plots (Supplementary Figure S2). Above all, the AsA D-Man/L-Gal pathway proteins were conserved in Brassicas.

### Divergence and Evolutionary History of the AsA D-Man/L-Gal Pathway Genes

The AsA D-Man/L-Gal pathway genes may have functioned in a few green algae plants and all higher plants (Urzica et al., 2012). To retain a stable network, these genes have been highly conserved and retained during evolution (Duan et al., 2015). However, the divergence of these genes is unclear. Among these genes in the AsA D-Man/L-Gal pathway of Brassicaceae plants, the number of genes that encode the same enzymes varied (Supplementary Table S3). The largest number of *GMP* genes was identified in *B. rapa* and was 2.5 times higher than that in *A. thaliana*. Phylogenetic analysis indicated that there were three clades (I, II, and III) of *GMP* genes in both species (Supplementary Figure S3A). Then, the microsynteny of all three clades genes were identified by comparing the *A. thaliana* and *B. rapa* genomes using CoGE (short for comparative genomics; Supplementary Figure S3B), which indicated that all of these *A. thaliana* genes, except for the *AT3G55590* locus, were duplicated in the *B. rapa* genome via triplication and then expanded in each clade. By analyzing the protein structure with MEME, we found a clear structural divergence among these three clades, particularly between clade I and clade III (Supplementary Figure S3C). In clade II, there was also a structural divergence among the paralogs. Here, we mainly focused on the *VTC1* gene in *B. rapa* because of its important role in the AsA D-Man/L-Gal pathway. After the triplication event, the *BraVTC1s* were all retained. However, the homologous genes did not have the same expression pattern; *BraVTC1a* was not expressed at a detectable level in these four tissues (Supplementary Figure S3D) (Duan et al., 2015).

**FIGURE 2:**
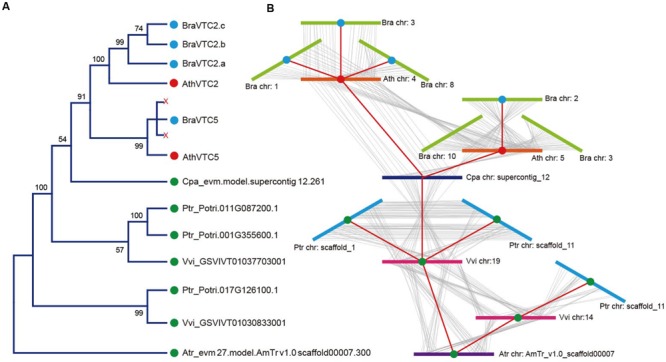
**Microsynteny analysis of the *GGP* genes in representative eudicots.** The following parts are shown from left to right. **(A)** Protein maximum likelihood (ML) tree: The tree was constructed by ML, and bootstrap values were calculated with 1,000 replications. The protein sequences used for the tree were listed in Supplementary Table S7. **(B)** Microsynteny analysis: the microsynteny of the GGPs in *A. thaliana, A. trichopoda, C. papaya, P. trichocarpa, V. vinifera* and *B. rapa* was evaluated with the CoGe comparative genomic tool (see in Supplementary Table S5) and indicated a possible GGP collinear relationship. *BraVTC2* retained three copies and *BraVTC5* lost two copies during fractionation.

*Arabidopsis thaliana* GGP is encoded by a pair of homologous genes, *VTC2* and *VTC5* (Dowdle et al., 2007), but in *B. rapa*, GGP is encoded by four genes. To refine the timing and relationship of the duplication, we investigated microsynteny in the regions flanking these genes. *A. trichopoda* has a single GGP loci; whereas *V. vinifera, P. trichocarpa, C. papaya, A. thaliana*, and *B. rapa* have two, three, one, two and four GGP loci, respectively, as a result of the duplications (Supplementary Table S5). By analyzing the phylogenetic tree and microsynteny of these genes (**Figures 2A,B**; Supplementary Table S6), we found that the duplication and fractionation of the *GGP* genes were different in these six species. There were two copies of the GGP locus in *V. vinifera* because of the γ duplication, but only one copy in *C. papaya*. In addition, one *GGP* gene was lost in *P. trichocarpa* during a salicoid duplication event (Tuskan et al., 2006). In *A. thaliana*, two copies of GGP, *VTC2* and *VTC5*, were retained after the duplication. Then, after triplication and fractionation in *B. rapa*, three copies of *VTC2* were retained, but only a single copy of *VTC5* was retained. From the microsynteny of the flanking genes, it was clear that these genes were all in the GGP syntenic position. We constructed a possible collinear relationship between the *GGP* genes among these species (**Figure 2B**). The *GGP* genes belong to a monophyletic group; *VTC5* and *VTC2* were not the ancestor clades in all angiosperms and were derived from a Brassicaceae-specific duplication event.

The numbers of most AsA D-Man/L-Gal pathway genes are not significantly different among plant species (Duan et al., 2015). We used phylogenetic analysis to evaluate the evolutionary history of GPP (*VTC4*) in twenty selected species (Supplementary Figure S4A). On the basis of sequence similarity, most of these genes were closely conserved. Through a protein structure analysis by MEME, we found that the VTC4s had highly conserved domains among these species, except for *BraVTC4*, which has a longer sequence than the others. In addition, the *A. thaliana, A. lyrata* and *B. rapa VTC4* sequences were further studied by using multiple sequence alignments (Supplementary Figure S4B). We found that the full-length sequences of both the *A. thaliana* and *A. lyrata VTC4* genes had a high degree of similarity with the 270 bp sequence of *BraVTC4*. In addition, we also investigated the microsynteny of *VTC4* in *A. thaliana* and *B. rapa* by CoGE and found that only one *B. rapa VTC4* gene was retained (Supplementary Figure S4C). Interestingly, two *A. thaliana* genes corresponded to one *B. rapa* gene; Bra040607 (*BraVTC4*) may be the combination of two genes, i.e., AT3G02870 (*AthVTC*4) and AT3G02860, according to the characteristics of the gene structures. However, this phenomenon does not appear in *B. oleracea* and *B. napus* (Supplementary Table S4). We speculate that Bra040607 arose from incomplete genome assembly or annotation errors, but these data do not permit us to distinguish between these two possibilities.

### FLC Influenced the AsA D-Man/L-Gal Pathway Gene VTC5 in NHCC

**FIGURE 3:**
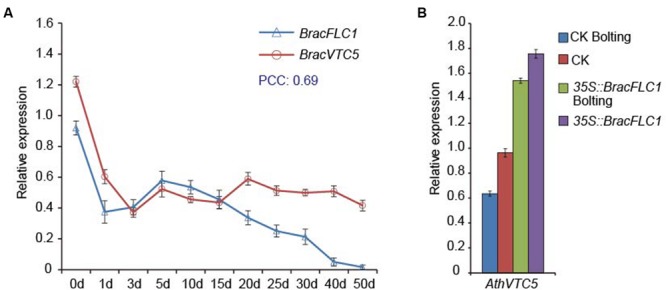
**Regulatory relationships between the *VTC5* and *FLC* genes based on their expression patterns. (A)** Expression patterns of *BracVTC5* and *BracFLC1* in *Brassica* leaves under simulated vernalization for 50 days. PCC indicates the Pearson’s correlation coefficients of the gene pairs using the transformed qRT-PCR data. **(B)** Expression of *AthVTC5* in T_2_ *BracFLC1* transgenic and CK *Arabidopsis* leaves. The transgenic *Arabidopsis* plants were 4 weeks old (vegetative) and 7 weeks old (bolting) and the CK plants were 4 weeks old (vegetative) and 6 weeks old (bolting). The T_2_ *BracFLC1* transgenic plants were from the study by Liu et al. (2012). The error bars **(A,B)** represent the standard errors from three independent replicates.

From ChIP-seq analysis, the potential *FLC* target genes have been defined by Deng et al. (2011), and *VTC5* (AT5G55120) is one of the putative *FLC* targets (Deng et al., 2011). In this study, to verify whether *VTC5* interacted with *FLC*, we detected the expression patterns of *BracFLC1* and *BracVTC5* in *Brassica* crop NHCC under simulated vernalization. Both genes were down-regulated in response to the 4°C treatment for 3 days. *BracFLC1* was slowly up-regulated and then continuously down-regulated, whereas *BracVTC5* was relatively stable (**Figure 3A**). Co-expression between these genes was established on the basis of the PCC by using the transformed qRT-PCR data, and the PCC value between them was 0.69. The results indicated that *FLC* may induce the expression of *VTC5*. To further verify this observation, we selected the overexpressed *BracFLC1* transgenic *A. thaliana* plants from the study by Liu et al. (2012). These transgenic plants of the T_2_ generation were confirmed by qRT-PCR; three lines (named T1, T2, and T3) with high expression of *BracFLC1* were verified (Supplementary Figure S5), and the vegetative and bolting stages were chosen for this experiment. The expression of *AthVTC5* decreased after the bolting of the CK plants. In addition, *BracFLC1* overexpression affected the expression of *AthVTC5*. *AthVTC5* expression was significantly increased in the transgenic plants (**Figure 3B**). These results indicate that *FLC* directly or indirectly affects the expression of *VTC5*.

### AsA D-Man/L-Gal Pathway Genes Are Regulated by Light in *B. rapa*

**FIGURE 4:**
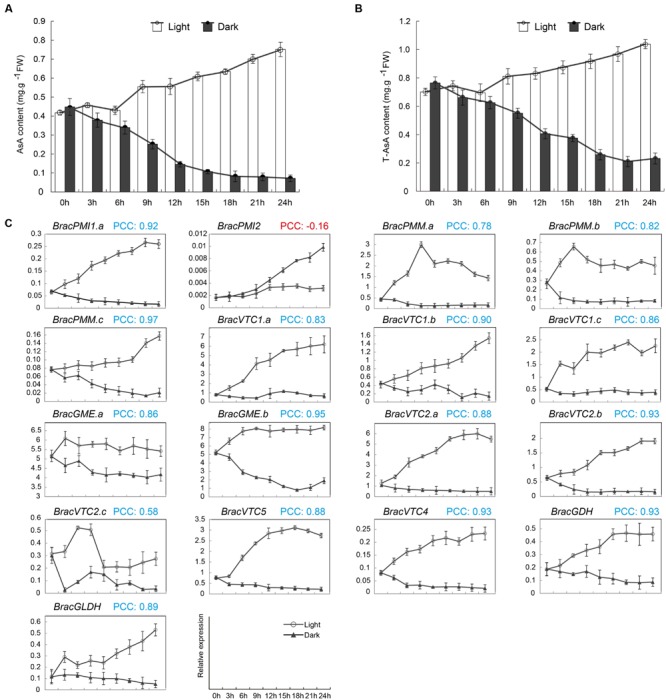
**Effects of light on the AsA levels and the expression of enzymes in the AsA biosynthetic pathway in *Brassica* crop NHCC leaves.** Five-leaf stage NHCC plants grown under 16 h of light and 8 h of darkness were moved into the dark or exposed to continuous light. The analysis started after 8 h of darkness (corresponding to the time of the beginning of the light period), as detailed in the Materials and methods. The leaves were extracted at the indicated times and assayed to determine the AsA, T-AsA and corresponding gene expression levels. **(A)** AsA and **(B)** T-AsA levels. **(C)** Transcript levels of enzymes of the AsA D-Man/L-Gal pathway. For each sample, the transcript levels were normalized to those of actin (control). PCC indicates the Pearson’s correlation coefficients between each light/dark-inducible gene using the transformed qRT-PCR data and the corresponding AsA content. The PCCs were considered significant at the 0.05 significance level (*p*-value). The data shown in **(A–C)** are the mean values ± SD of three individual experiments (*n* = 3).

It has been shown that leaves exposed to high light contain more AsA compared with those in the shade. The AsA content during the day is different depending on the time, although it is higher between 12:00 to 14:00 than at any other time period (Massot et al., 2012). In addition, the AsA content was regulated in leaves, probably protecting the plant against photo-oxidative stress. However, in *B. rapa*, the regulation of the AsA D-Man/L-Gal pathway gene expression levels may be more complex than in *Arabidopsis* because several copies code for the same enzyme. To explore the light-mediated regulation of AsA biosynthesis in *B. rapa*, 24 h continuous light and dark treatments of NHCC were used. After 24 h in the darkness, the AsA levels in the leaves decreased by 90%. However, the level was increased by 180% after the plants were exposed to continuous light for 24 h (**Figure 4A**). Moreover, the T-AsA levels in the leaves showed the same trend as the AsA levels in both the light and dark treatments (**Figure 4B**). To understand the mechanism by which the leaf AsA levels decrease in darkness, the levels of transcripts of homologous genes involved in the AsA D-Man/L-Gal pathway were verified (**Figure 4C**). With the exception of *BracPMI2*, the expression patterns of all genes were down-regulated in the dark. Furthermore, the transcript level of *BracGME.b* was more rapidly decreased under continuous darkness compared with the other genes. *BracPMI2*, which exhibited clear anomalies compared with *BracPMI1.a* (**Figure 4C**), was activated under darkness, similarly to *AtPMI2*, which is induced by only a long period in the dark (Maruta et al., 2008). In continuous light, in contrast to darkness, the transcript levels of all genes showed varying degrees of regulation.

**FIGURE 5:**
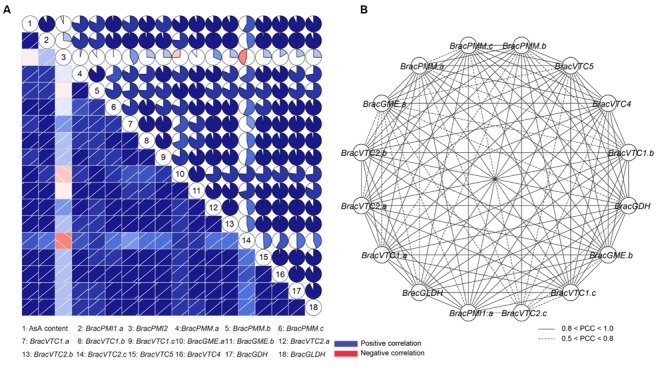
**Correlations between the AsA contents and the transcript levels of enzymes of the AsA D-Mannose/L-Galactose pathway and co-regulatory networks of the light/dark-inducible genes. (A)** Correlation analysis with the R package. The PCCs were calculated on the basis of the total expression trends in response to continuous light and continuous darkness. Lower squares: the correlations are indicated by the color and intensity of shading. Upper squares: the correlations are indicated by circular symbols. Each correlation is shown by shades of blue and red and the size of the fan shape. Blue and red indicate positive correlation and negative correlation, respectively. Different numbers represent the AsA content and the different genes. **(B)** Co-regulatory networks. The co-regulatory networks of light/dark-inducible AsA biosynthetic genes were established on the basis of the PCCs of the gene pairs by using the transformed qRT-PCR data, which included 16 nodes and 115 regulatory edges. The PCCs of the co-regulated gene pairs was considered to be significant at the 0.05 significance level (*p*-value), and the different edge line styles indicate the different significance levels of the co-regulated gene pairs.

To understand the connection between the transcript levels of the AsA biosynthesis-related genes and the AsA levels, co-regulatory networks were established on the basis of the PCCs of the AsA content and the relative expression of these genes (**Figure 5A**). Clearly, with the exception of *BracPMI2*, a high correlation between all of these genes and the AsA content was determined. Furthermore, a similar expression pattern was identified in the homologous genes in response to the light/dark treatments (**Figure 5A**). More interestingly, the enzyme genes in this biosynthetic network, whose products may be sensitive to the light/dark treatments, showed similar transcriptional patterns (**Figure 5A**). All PCCs that were significant at the 0.05 significance level (*p*-value) were collected and visualized with the Cytoscape program to construct light-related co-regulatory networks of the AsA D-Man/L-Gal pathway genes (**Figure 5B**). There were 16 nodes representing the light/dark treatments connected by 115 edges, which represented the PCCs of the co-regulated gene pairs (**Figure 5B**; Supplementary Table S7). Most of the (92/115) co-regulated gene pairs appeared to have significant positive correlations (PCC > 0.8), thus indicating their important roles in responding to the light/dark treatments.

### Expression Patterns and Co-regulatory Networks of the AsA D-Man/L-Gal Pathway Genes in *B. rapa* in Response to Multiple Stresses

**FIGURE 6:**
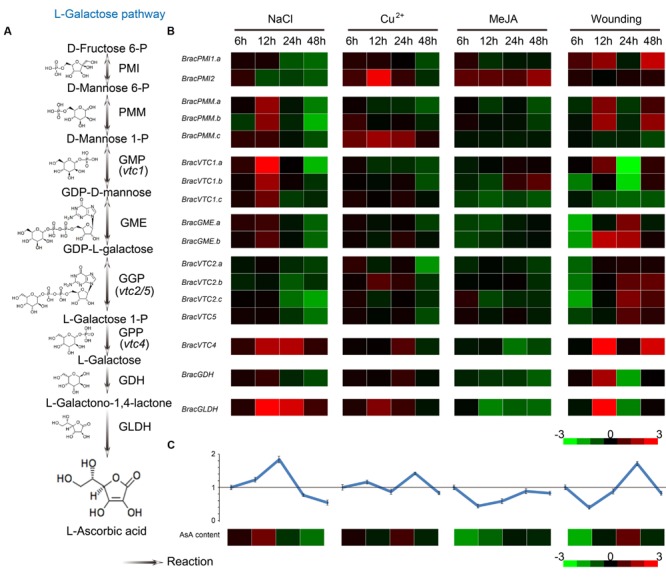
**Expression of AsA biosynthetic genes in *Brassica* crop NHCC leaves in response to multiple stresses. (A)** The primary AsA D-Mannose/L-Galactose pathway biosynthetic routes. **(B)** Analysis of the transcript levels of the AsA biosynthetic genes. NHCC plants were subjected to NaCl, Cu^2+^, MeJA and wounding treatments. The expression of the AsA biosynthetic genes was significantly increased in the leaves in response to multiple abiotic stresses, and their transcript levels were normalized to the actin transcript levels with the 2^-ΔΔCT^ method (see Supplementary Table S9). **(C)** AsA levels in response to multiple stresses. For each sample, the AsA levels were normalized to the CK levels. All experiments were performed in triplicate. The heat maps shown in B and C were constructed using Java TreeView. The bar at the bottom of each heat map represents the relative values.

To investigate the effects of various stress conditions on the expression of AsA biosynthetic enzymes and to discern the key regulatory genes, the effects of four stresses [NaCl, Cu^2+^, methyl jasmonate (MeJA) and wounding] on the AsA D-Man/L-Gal pathway genes were explored (**Figure 6**). The divergence of the expression patterns for the homologous genes was also identified. The changes in the AsA levels in response to these four stresses were diverse, suggesting that there were different degrees of demand for antioxidants to compensate for the presumed increased amounts of ROS. Specifically, the AsA content in the leaves was increased in response to NaCl before 12 h and then decreased. Under Cu^2+^ and wounding stresses, the levels were increased at 24 h; under MeJA stress, they were continuously decreased (**Figure 6**). To further understand the mechanism of diversification in the leaf AsA levels in response to these stresses, the transcript levels of the homologous genes were also verified. The expression of most biosynthetic genes was induced in NHCC leaves in response to these stresses (**Figure 6B**). In particular, most genes were up-regulated by NaCl stress at 12 h, a result consistent with the changes in the AsA content. However, most genes were down-regulated in response to MeJA stress, except for *BracPMI2*. These changes may have led to the decreased AsA content in response MeJA stress. Under Cu^2+^ and wounding stresses, the expression patterns of only some genes were consistent with the AsA content. The increased ROS production in response to Cu^2+^ and wounding stresses suggests that these ROS detoxification systems may be complex and that the metabolic processes responding to the antioxidant systems varied. Overall, the expression patterns of the genes that encode the GME and GPP enzymes were fairly consistent with the AsA content in response to these four treatments. We speculate that these two enzymes have a major role in regulating and limiting AsA biosynthesis in *B. rapa* in response to multiple abiotic stresses.

To clearly understand the connection between the transcript levels of these genes and the AsA content, co-regulatory networks were also established on the basis of the PCCs of the AsA content and the relative expression trends of the genes (Supplementary Figure S6A). We also examined the divergence of the homologous genes. With the exception of the *PMI* genes, all of the AsA D-Man/L-Gal pathway genes appeared to have different degrees of positive correlations (Supplementary Figure S6A). Consistently with previous conclusions, the *GME* and *GPP* genes appeared to have significant positive correlations, particularly *BracGME.a*, which had a PCC of 0.9. Furthermore, the upstream and downstream enzyme genes were found to have positive correlations, except for *BracPMI2* vs. *BracPMI2* and *BracVTC5* vs. *BracVTC4* (Supplementary Figure S6A). Then, all PCCs that were significant at the 0.05 significance level (*p*-value) were collected and visualized by the Cytoscape program to construct the stress co-regulatory networks of these genes. We connected 40 edges to represent these gene nodes under multiple stresses, which were significantly fewer than those under the light/dark treatments (Supplementary Figure S6B; Supplementary Table S8). This observation may be due to light-responsive promoter motifs that were found in a high percentage of AsA-related genes (Ioannidi et al., 2009), as well as the complex ROS detoxification systems induced by multiple stresses. Most importantly, the genes in the AsA D-Man/L-Gal pathway were not expressed under normal growth conditions (Supplementary Figure S6C) and possessed different transcript levels that corresponded to the complex environments (**Figure 6**).

## Discussion

The D-Man/L-Gal pathway is a significant source of ascorbate, on the basis of biochemical and molecular genetic evidence. Overexpression of the genes in this pathway increases the AsA content to varying degrees in plants (Cruz-Rus et al., 2012). In contrast, antisense inhibition of these genes leads to decreased AsA content in plants (Conklin et al., 1999), thus indicating the important roles of these genes in AsA biosynthesis. However, too much or too little AsA damages plants to different degrees. A global alteration in the AsA content may have undesired deleterious effects because of the key role of AsA in some plant developmental processes and stages (Cruz-Rus et al., 2012). Thus, for different species, AsA has a certain genetic stability. Moreover, a pattern of evolutionary conservation of AsA after WGT has been observed in *B. rapa* (Duan et al., 2015).

In the plant kingdom, higher plant lineages have undergone polyploidization during their long evolutionary history. WGD events have been important for the evolution of complexity in multicellular eukaryotes (Edger and Pires, 2009). During these events, the genes that are highly connected within metabolic networks exhibit preferential retention, as has been confirmed in *Arabidopsis* (Bekaert et al., 2011). AsA-related genes in biosynthetic networks also conform to the gene balance hypothesis (Duan et al., 2015). To date, the genomes of the ‘U’s triangle *Brassica* crops: *B. rapa, B. oleracea*, and *B. napus* have been sequenced and assembled. The *B. oleracea* genome has undergone triplication events since its divergence from *Arabidopsis*, and *B. napus* was formed by hybridization between *B. rapa* and *B. oleracea*, followed by chromosome doubling (Chalhoub et al., 2014; Liu S. et al., 2014). In this study, we found that the AsA D-Man/L-Gal pathway genes were preferentially retained in *B. rapa* compared with their neighboring genes, and similar retentions were identified in the *Brassica* AA and CC genomes. The orthologs of these genes had similar intron and exon numbers, indicating they may have similar gene structures. Furthermore, a high degree of sequence similarity (>95%) remained among these species. We inferred that the AsA D-Man/L-Gal pathway genes were preferentially retained, owing to their important roles in protecting plants against oxidative damage. However, their evolutionarily conservation occurred because the AsA content must be tightly regulated in plants. This conservation would be expected, given the important and diverse roles of AsA in plants, as evidenced primarily by the moderate effects on the AsA content in genetically modified plants (Cruz-Rus et al., 2012).

Although these genes have high conservation and retention, functional differentiation would allow them to better adapt to their environment. Polyploidy has led to WGD and has provided opportunities for duplicated genes to diverge in several ways during evolution. Each of these genes could subsequently follow one of three broad fates: subfunctionalization, neofunctionalization, and non-functionalization (deletion or pseudogenization) (Innan and Kondrashov, 2010). In this study, we found that there were three major types of divergence in these AsA-related genes: divergence of subclades of multigene families, divergence of paralogous genes, and conservation of gene copies. *GMP* belongs to a multigene family composed of three clades. Different structural and expression patterns were observe in the three clades. Moreover, some genes are not unique to AsA biosynthesis, but they are responsible for the biosynthesis of nucleotide sugar, which serves as the substrate for AsA biosynthesis and as a precursor for cell wall polysaccharides and glycoproteins. This phenomenon may result in the divergence of multigene family genes. *PMI* has two homologous genes. *PMI1* was constitutively expressed in various tissues of *Arabidopsis* under normal growth conditions, and its gene expression was induced by continuous light. However, the expression of *PMI2* is induced by only a long period in the dark (Maruta et al., 2008). This result is consistent with the expression pattern of *PMI* in *B. rapa*. We inferred that this duplication conforms to the neofunctionalization mode, in which one duplicate copy accumulates beneficial mutations and acquires a new function (Li et al., 2005). Moreover, the *GGP* homolog *VTC5* also conformed to the neofunctionalization or subfunctionalization mode because it may have acquired a new function, thus becoming one of the putative *FLC* targets. *VTC5* is influenced by *FLC* and is co-expressed during plant bolting. However, this influence has not been identified in *VTC2* (Supplementary Figure S7). In addition to these genes, some genes were conserved, such as *GPP, GDH* and *GLDH*, with only a single copy being identified in most plant species. Because of these characteristics, plants are expected to adapt to a more complex environment under the premise of ensuring stability.

We conclude that the AsA D-Man/L-Gal pathway genes exhibit both conservative and expression patterns divergence. Furthermore, under normal circumstances, the leaf AsA levels depend on the light/dark conditions (Tabata et al., 2002). It is well known that leaf AsA levels show a diurnal rhythm (Dutilleul et al., 2003; Tamaoki et al., 2003) and vary with the daylight conditions (Grace and Logan, 1996). This dependence may be due to light-responsive promoter motifs that have been identified in the AsA-related genes (Ioannidi et al., 2009). In *Arabidopsis, VTC2* (Müller-Moulé, 2008), *PMI* (Maruta et al., 2008), *GalDH* (Gatzek et al., 2002) and *GalLDH* (Tamaoki et al., 2003) have already been shown to be regulated by light. In *B. rapa*, a substantial correlation was observed between the AsA D-Man/L-Gal pathway genes and the AsA content in response to the light/dark treatments in this study. These genes are involved in the pathway in which the light response regulates the AsA levels. With the exception of the *PMI* genes, the homologous genes appeared to have a similar expression pattern and positive correlations with light. This pattern underscores the importance of light in the regulation of the biosynthesis of AsA, particularly in *B. rapa*.

In the external environment, the plants will be subjected to various abiotic stresses, thus leading to survival of the fittest in their evolutionary history. One of the most important proposed roles for AsA is as a response to many stresses, including oxidative processes (Noctor and Foyer, 1998; Smirnoff and Wheeler, 2000). Its protective effects against photo-oxidative stress and ozone have been well documented (Sanmartin et al., 2003; Müller-Moulé et al., 2004). Multiple abiotic stresses can lead to variations in ROS production and the induction of ROS detoxification systems. In *B. rapa*, the high *PMI, GME*, and *GGP* expression levels at least partially contribute to the increased AsA accumulation, as determined by a comparison of the AsA genes in different tissues of three NHCC cultivars (Ren et al., 2013). Moreover, the expression patterns of the genes that encode the GME and GPP enzymes were also consistent with AsA content under the four stresses (NaCl, Cu^2+^, MeJA and wounding). We speculate that these two enzymes have a major role in regulating and limiting AsA biosynthesis in response to multiple abiotic stresses. Other genes have different levels of expression in response to multiple abiotic stresses. Some of these genes are not expressed under normal growth conditions (Supplementary Figure S6C) (Duan et al., 2015). This finding suggests that the decreased expression of some genes may be an adaptive response to a dosage rebalances, although it is probably neutral in most other cases. It has been proposed that functionally redundant duplicate genes may serve as a backup for important functions in the event of a severe mutation, much like a spare tire in a car (Qian et al., 2010). Here, we term this scenario the backup hypothesis. In our experiment on the expression patterns of AsA biosynthesis-related genes, we found interesting results supporting this hypothesis. For example, *PMM3* expression is almost undetectable in the leaves of the three NHCC cultivars (Ren et al., 2013). However, the relative expression of *PMM3* was up-regulated in response to multiple abiotic stresses, particularly Cu^2+^ stress.

## Conclusion

**FIGURE 7:**
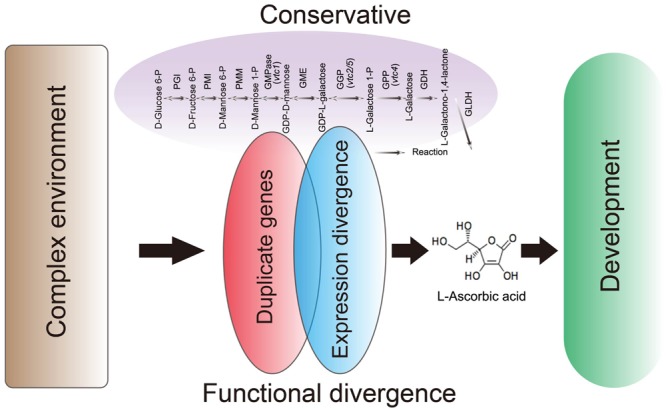
**Proposed model for the conservation and functional divergence of the AsA D-Mannose/L-Galactose pathway genes in *Brassica rapa***.

In our study, we present evidence indicating that the conservation of D-Man/L-Gal pathway genes maintains a suitable level of AsA in plants through similar retention and a high degree of sequence similarity of these genes in both the AA and CC *Brassica* genomes. In addition, the expression data in NHCC subjected to different treatments suggest that the number of functional, duplicate gene copies within these genes varies. Our results strongly suggest that environmental changes modulate the transcriptome for fine regulation of AsA biosynthesis (**Figure 7**). Moreover, some genes are not expressed under normal growth conditions, but function under stress treatments, thus supporting the backup hypothesis. Moreover, GME and GPP play key roles in this pathway by regulating and limiting AsA biosynthesis in *B. rapa* in response to multiple abiotic stresses. Our findings show that the conservative and expression patterns divergence of D-Man/L-Gal pathway genes not only avoids AsA biosynthesis network instability but also allows *B. rapa* to better adapt to complex environments.

## Author Contributions

The study was conceived by WD. WD collected the public datasets for Brassicas and the other researched species. WD, TL, ZC, and XS contributed to data analysis, bioinformatics analysis and manuscript preparation. WD, JR, ZH, and YL participated in the qRT-PCR experiment. YL and XH participated in the planning of experiments and revising the manuscript. All authors read and approved the final version of the manuscript.

## Conflict of Interest Statement

The authors declare that the research was conducted in the absence of any commercial or financial relationships that could be construed as a potential conflict of interest.

## Footnotes

1 http://brassicadb.org/brad/

2 http://www.ocri-genomics.org/bolbase/

3 http://www.genoscope.cns.fr/brassicanapus/

4 https://phytozome.jgi.doe.gov/pz/portal.html

5 http://www.ncbi.nlm.nih.gov/

6 http://brassicadb.org/brad/searchSynteny.php

7 http://www.genomevolution.org/CoGe/GEvo.pl

8 http://chibba.pgml.uga.edu/mcscan2/

## Abbreviations

GDH: L-galactose dehydrogenase
GGP: GDP-L-galactose phosphorylase
GLDH: L-galactono-1, 4-lactone dehydrogenase
GME: GDP-mannose-3′, 5′-epimerase
GMP: GDP-mannose pyrophosphorylase (mannose-1-phosphate guanyltransferase)
GPP: L-galactose-1-phosphate phosphatase
ML: maximum-likelihood
NHCC: non-heading Chinese cabbage
PCCs: Pearson’s correlation coefficients
PGI: phosphoglucose isomerase
PMI: D-mannose-6-phosphate isomerase
PMM: phosphomannomutase
qRT- PCR: quantitative real-time PCR
ROS: reactive oxygen species
VTC: vitamin C
