# Supplementary material for: Conservation and Expression Patterns Divergence of Ascorbic Acid d-mannose/l-galactose Pathway Genes in Brassica rapa
Source: Front Plant Sci. 2016 Jun 2;7:778. doi: 10.3389/fpls.2016.00778 (PMC4889602; doi:10.3389/fpls.2016.00778)
Supplement: Supplementary file 2 [file Image_1.PDF]

Supplementary Material

Conservative and functional divergence of ascorbic acid D-mannose/L-galactose pathway genes in *Brassica rapa*

Weike Duan[1], Jun Ren[1,], Yan Li[1], Tongkun Liu[1], Xiaoming Song[1, 2], Zhongwen Chen[1], Zhinan Huang[1], Xilin Hou[1], Ying Li [1*]

[1]State Key Laboratory of Crop Genetics and Germplasm Enhancement, Key Laboratory of Biology and Germplasm Enhancement of Horticultural Crops in East China, College of Horticulture of Nanjing Agricultural University, Nanjing 210095, P.R. China
[2]Center of Genomics and Computational Biology, College of Life Sciences, North China University of Science and Technology, Tangshan, Hebei 063000, P.R. China.

Correspondence:
Prof: Ying Li
yingli@njau.edu.cn

Supplementary Figure 1-7

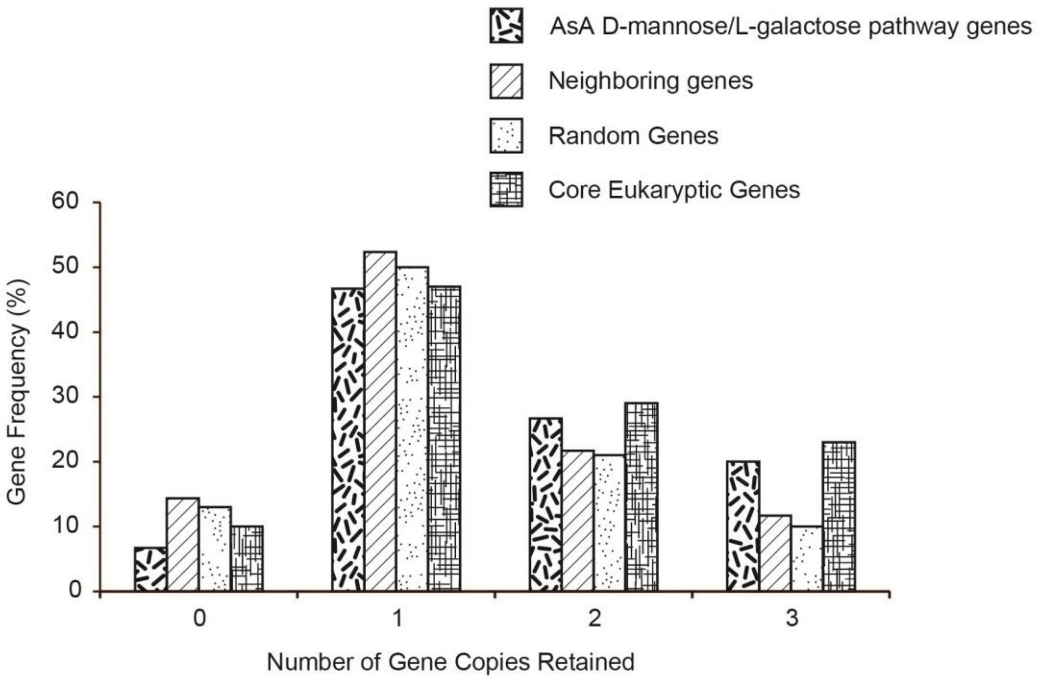

Fig. S1. Retention rates of AsA D-mannose/L-galactose pathway genes with the neighboring, randomly selected, and core eukaryotic genes in the syntenic region of them after genome triplication and fractionation in *Brassica rapa*.

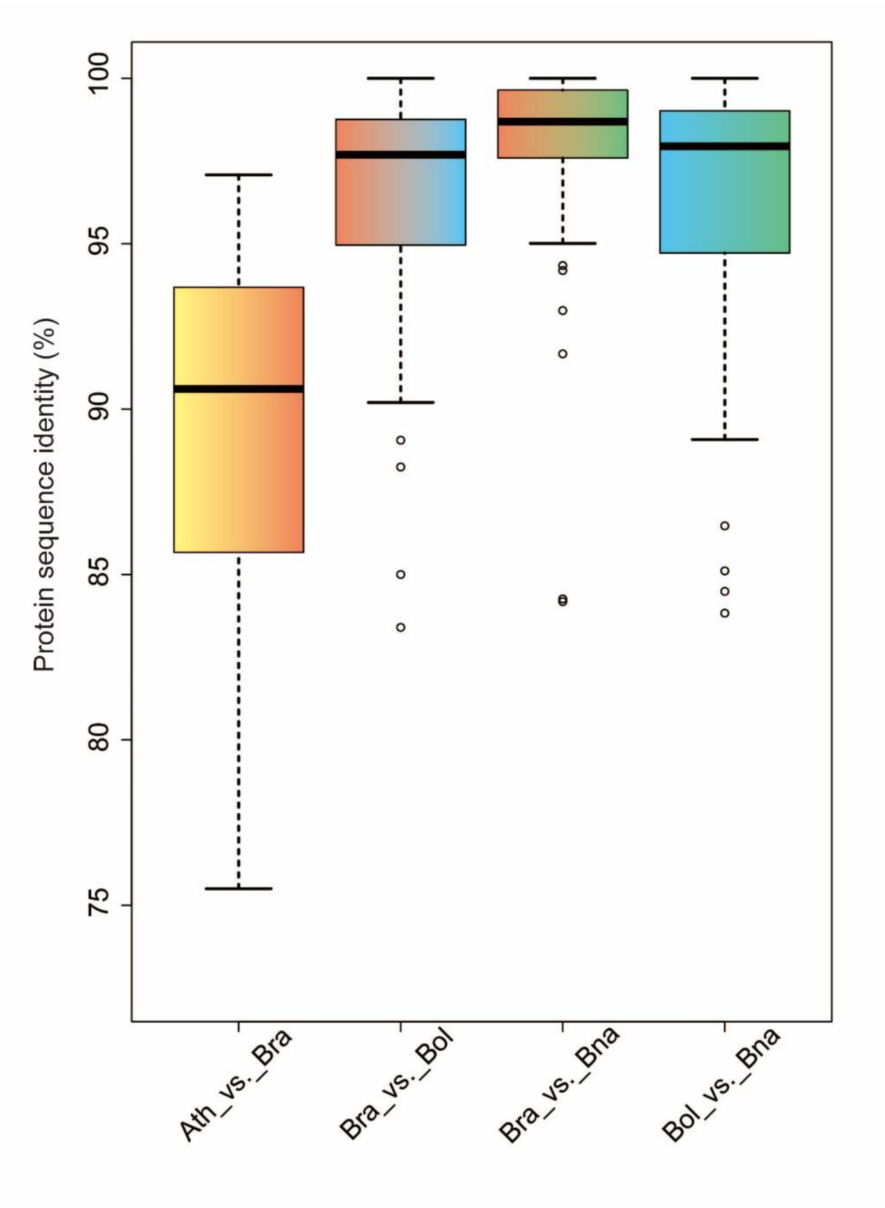

Fig. S2. Pairwise sequence identity of full-length AsA D-mannose/L-galactose (D-Man/L-Gal) pathway proteins. Ath, Bra, Bol and Bna represent *Arabidopsis thaliana*, *Brassica rapa*, *Brassica oleracea* and *Brassica napus*, respectively. Vs. represents pairwise sequence identities between both species. The box plot shows the median (black line), interquartile range (box), and maximum and minimum scores (whiskers) of each data set.

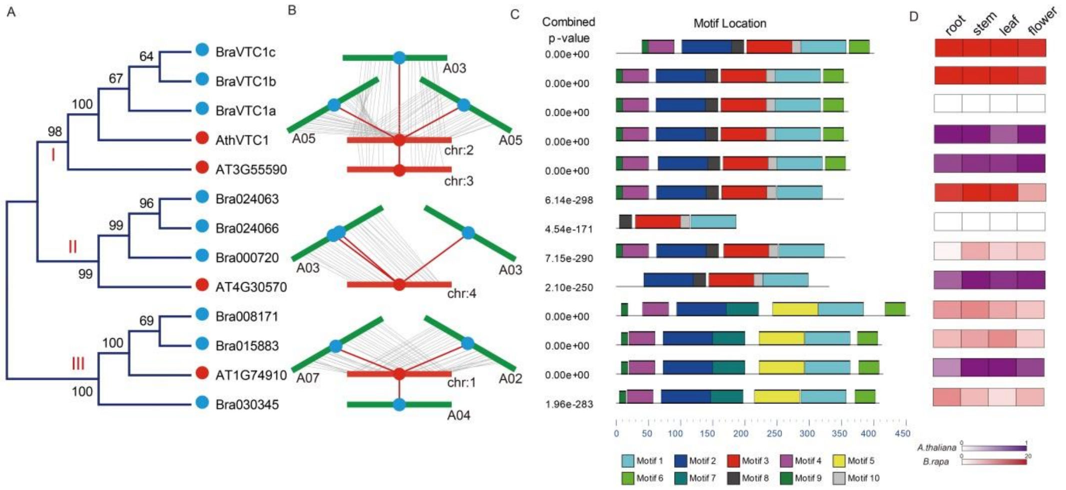

Fig. S3. An analytical view of the *GMP* gene family in *Arabidopsis thaliana* and *Brassica rapa*. The following parts are shown from left to right. (A) Protein maximum likelihood (ML) tree: The tree was constructed by ML and bootstrap values were calculated with 1000 replications. (B) Microsynteny analysis: Microsynteny analysis of the GMP genes in *A. thaliana* and *B. rapa* by CoGE (see in Table S5). (C) Protein structure: Each colored box represents a conservative motif. (D) Expression pattern: The gene expression in the four tissues (root, stem, leaf and flower) was constructed as described Duan et al. (2015).

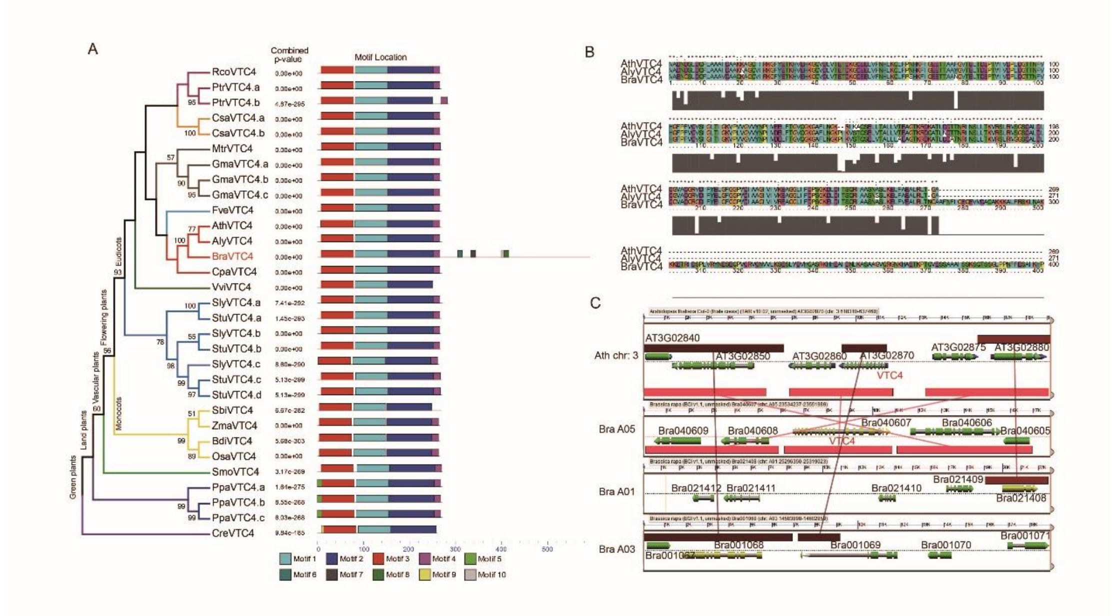

Fig. S4. An analytical view of the *GPP* genes. (A) The following parts are shown from left to right. Protein maximum likelihood (ML) tree: The tree was constructed by ML among selected twenty plant species and bootstrap values were calculated with 1000 replications. The GPP homologs in the tree was constructed as described in Table S4. Protein structure: Each colored box represents a conservative motif. The used protein sequences were list in Table S4. (B) Multiple sequence alignment of *VTC4* genes among *A. thaliana*, *A. lyrata* and *B. rapa*. (C) Microsynteny analysis of the *VTC4* genes in *A. thaliana* and *B. rapa* by CoGE (see in Table S5).

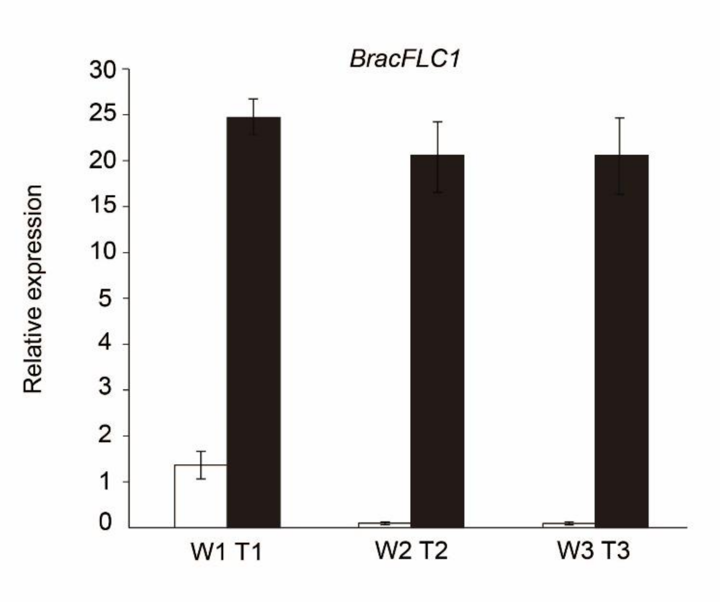

Fig. S5. Expression of *BracFLC1* in T_2 transgenic and WT *Arabidopsis* leaves. W1, W2 and W3, wild-type plants at vegetative, bolting and flowering stages, respectively; T1, T2 and T3, transgenic plants at vegetative, bolting and flowering stages, respectively. Data are the mean values ±SD of three individual experiments (n = 3).

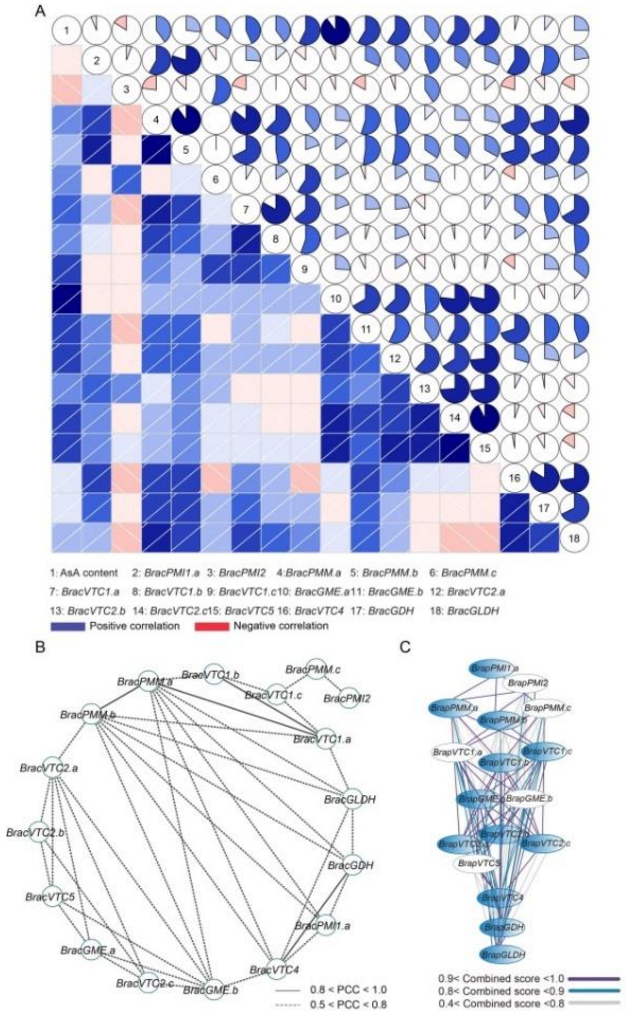

Fig. S6. Correlation among AsA content and transcript levels of enzymes of the AsA D-Man/L-Gal pathway and co-regulatory networks of the multiple stress-inducible genes. (A) Correlation analysis by using R package corrgram. The PCCs were calculated based on the expression trends under the four stresses (NaCl, Cu^{2+}, methyl jasmonate (MeJA) and wounding) treatments. Lower squares: correlations showed by color and intensity of shading; upper: circle symbols. Each correlation is shown by the shades of blue and red and the size of the fan shape. Blue and red indicate positive correlation and negative correlation, respectively. Different numbers represent AsA content and the different genes. (B) Co-regulatory networks. The co-regulatory networks of multiple stresses-inducible AsA biosynthesis genes were established based on the PCCs, which involved 17 nodes and 40 regulatory edges. PCC of co-regulatory gene pairs were significant at the 0.01 significance level (p-value), and different edge line styles indicate the different significance levels of the co-regulated gene pairs. (C) The interaction network of AsA biosynthesis genes in *B. rapa* was based on the orthologs in *A. thaliana* under normal growth conditions according to our previous study (Duan et al. 2015). Blue ellipses indicates genes with high expression levels in leaves, and white indicates those with no or low expression in leaves.

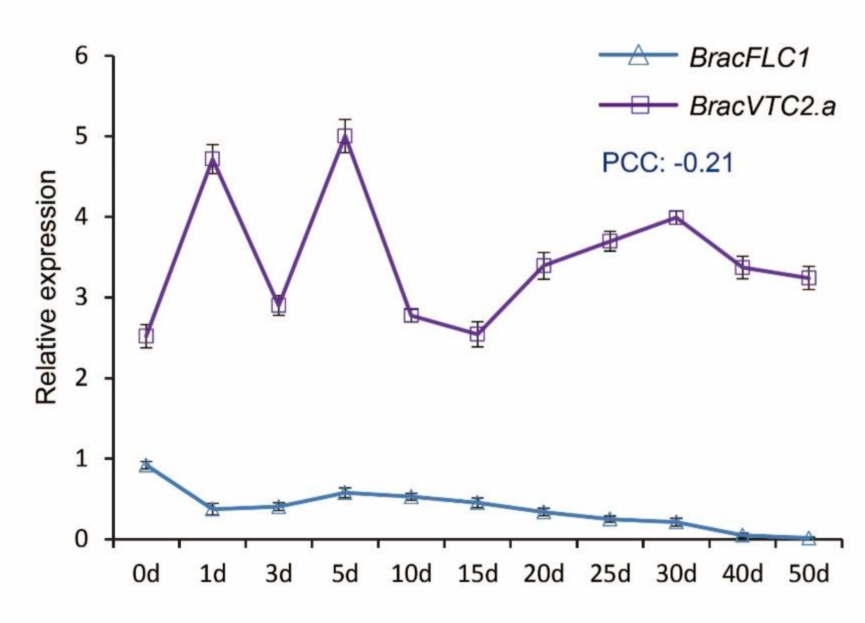

Fig. S7. Regulatory relationships of the *VTC2* and *FLC* genes based on their expression patterns. Expression patterns of *BracVTC2.a* and *BracFLC1* under simulated vernalization for 50 day in *Brassica* leaves. PCC indicates the Pearson correlation coefficients of the gene pairs using transformed qPCR data. Error bars in represent standard errors from three independent replicates.
